# Prompt engineering in medical education. Dissecting the new technological frontier in Digestive Surgery

**DOI:** 10.1590/0102-67202026000007e1936

**Published:** 2026-07-03

**Authors:** Ozimo Pereira GAMA, Breno Dias LIMA RIBEIRO, Paulo KASSAB

**Affiliations:** 1 Universidade Federal do Maranhão, Faculty of Medicine, Topographic Anatomy - São Luís (MA), Brazil.; 2 Universidade Federal do Maranhão, Faculty of Medicine, Department of Medicine - São Luís (MA), Brazil.; 3 Santa Casa of São Paulo, Medical School, Department of Surgery - São Paulo (SP), Brazil.

**Keywords:** Artificial Intelligence. Medical Education. Engineering. Ethics, Medical. Computer-Assisted Instruction, Inteligência Artificial, Educação Médica, Engenharia, Ética Médica, Instrução por Computador

## Abstract

Generative Artificial Intelligence (AI) has become a tangible reality in medical education, yet many struggle to interact effectively with these models. This editorial introduces Prompt Engineering as the modern “digital stethoscope”-a systematic approach to maximize AI potential. A structured prompt relies on four pillars (Context, Request, Persona, Format) and advances through four levels of complexity. Strategies like instructing the AI to “think step by step” mitigate logical errors in clinical tasks. However, users must remain vigilant against AI “hallucinations” and phantom citations by using verified databases. Ultimately, while AI processes vast data, human clinical judgment remains the irreplaceable filter for patient safety.

## ARTICLE HIGHLIGHTS

• The anatomy of a “perfect prompt” rests on four fundamental pillars.

• The most clinically relevant impact of advanced prompting is the mitigation of catastrophic errors.

• Ultimately, Prompt Engineering is more than a technical skill; it is a new pedagogy.

• The success of medical education lies not merely in the transfer of facts, but in forging a mind capable of interrogating reality with precision.

## VISUAL ABSTRACT

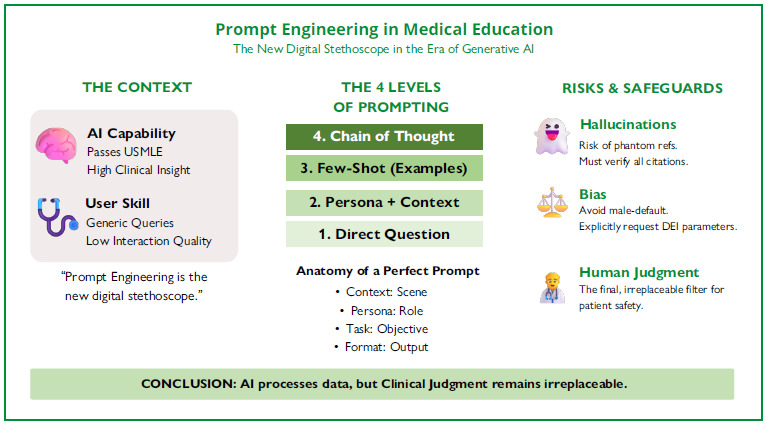

## RESUMO

A Inteligência Artificial (IA) Generativa tornou-se uma realidade tangível na educação médica, contudo, muitos ainda têm dificuldade em interagir de forma eficaz com esses modelos. Este editorial apresenta a Engenharia de Prompt como o “estetoscópio digital” moderno - uma abordagem sistemática para maximizar o potencial da IA. Um prompt estruturado baseia-se em quatro pilares (Contexto, Requisição, Persona, Formato) e avança por quatro níveis de complexidade. Estratégias como instruir a IA a “pensar passo a passo” mitigam erros lógicos em tarefas clínicas. No entanto, os usuários devem permanecer vigilantes contra as “alucinações” da IA e citações fantasmas, utilizando bancos de dados verificados. Por fim, embora a IA processe vastas quantidades de dados, o julgamento clínico humano permanece como o filtro insubstituível para a segurança do paciente.

## INTRODUCTION

Generative Artificial Intelligence (AI) has transitioned from a futuristic promise to a tangible academic reality, evidenced by its ability to perform at or above the threshold of the United States Medical Licensing Examination (USMLE) without specialized training. However, a paradox persists in medical schools: while AI demonstrates surprising clinical insight, most students and faculty have yet to master the “language” required to interact with these models effectively. To understand the qualitative leap of this tool, one must observe its technical evolution: GPT-1 (2018) operated with 117 million parameters, whereas modern models process trillions, changing the paradigm from simple indexing to complex knowledge synthesis. For the surgeon, this means AI has evolved from a medical dictionary into a logic consultant, demanding instructions as precise as the cleavage plane in an oncological dissection.

**Figure 1.**
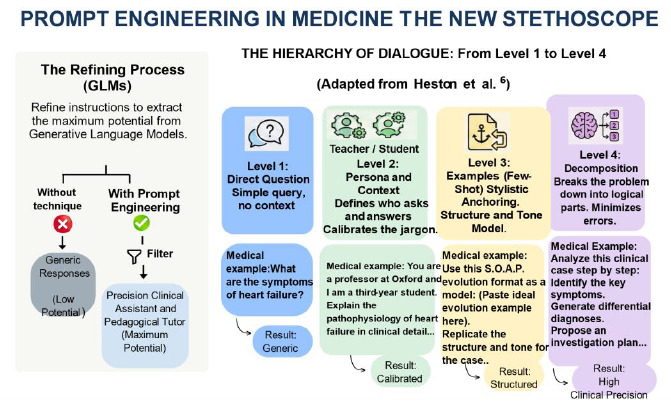
The perfect prompt.

In this context, Prompt Engineering emerges as the “digital stethoscope” of the modern era (Figure 1). As defined by Heston et al., it is a systematic approach to communicating with AI to obtain high-precision results, maximizing the potential of Generative Language Models (GLMs)^6^.

**Figure 2.**
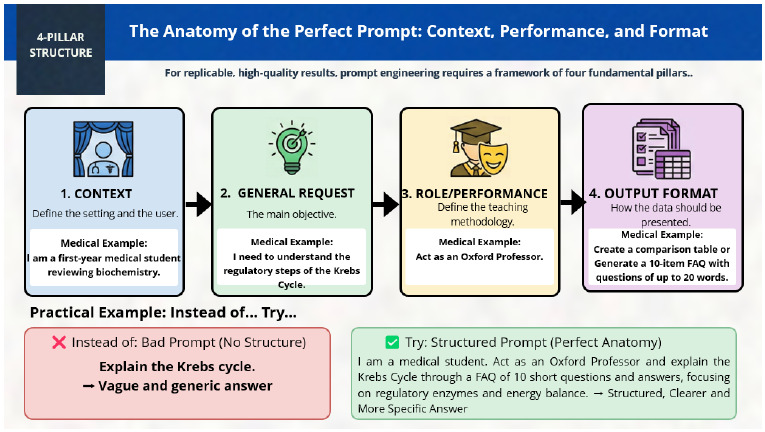
Prompt engineering.

The dialogue with AI is not a unidirectional query but a structured hierarchy of sophistication. The anatomy of a “perfect prompt” (Figure 2) rests on four fundamental pillars, much like the technical rigor required in a cholecystectomy: Context (setting the scenario), General Request (the core objective), Persona/Role (defining the behavior, such as a “Professor of Surgical Gastroenterology”), and Output Format (e.g., comparative tables or structured FAQs). Based on frameworks adapted for medical education, prompts can be scaled in complexity from Level 1 (Direct Question), Level 2 (Persona and Context), Level 3 (Few-Shot/Example-based, e.g., providing a clinical template for mesenteric ischemia), to Level 4 (Component Decomposition or “Chain of Thought”)^6^. Recent systematic reviews highlight that well-structured prompts are essential for generating high-quality multiple-choice questions (MCQs) and clinical vignettes, ensuring the AI functions as a precision pedagogical tutor^1^ ^,^ ^2^ ^,^ ^7^.

The most clinically relevant impact of advanced prompting is the mitigation of catastrophic errors. Instructing a GLM to “think step by step” utilizes its predictive nature to create an external “working memory,” significantly reducing errors in complex logical reasoning. In the surgical environment, this logic is what prevents iatrogenesis during the multi-level staging of gastric cancer or in complex pharmacological calculations^5^ ^,^ ^7^. Furthermore, AI can be used to simulate realistic “Virtual Patients”. For instance, generating a dynamic scenario to evaluate post-operative pain control strategies after bariatric surgery allows students to train clinical reasoning before real patient contact, echoing the trends observed in recent medical curricula^5^.

**Figure 3.**
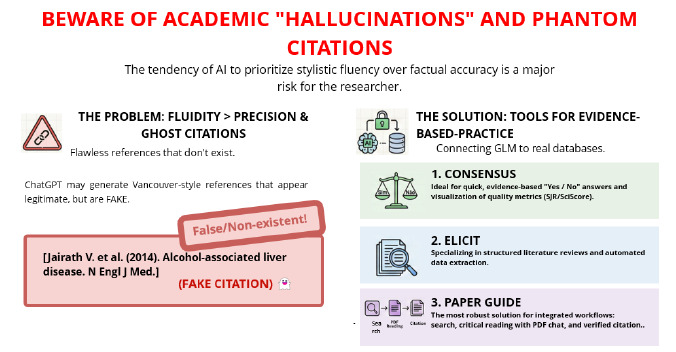
Hallucination prevention.

Despite its potential, the risk of “hallucinations” (Figure 3), where AI prioritizes stylistic fluency over factual accuracy, remains a critical concern for researchers. The generation of non-existent Vancouver-style citations (phantom citations) demands the integration of evidence-based tools (like Consensus or Elicit) and the strict verification of primary databases^3^ ^,^ ^8^. Furthermore, the integration of AI in medical education must address ethical imperatives. Poorly designed prompts can perpetuate biases. Therefore, instructions must explicitly include Diversity, Equity, and Inclusion (DEI) parameters, such as requesting cardiovascular or digestive cases in female patients to explore atypical presentations^5^ ^,^ ^8^.

In the Brazilian public health context, where high-stakes exams like The National Residency Exam (ENARE) and The National Medical Training Assessment Exam (ENAMED) demand extreme temporal efficiency - such as creating personalized mnemonics for acute pancreatitis severity criteria - the adoption of federated learning and AI-assisted reviews provides vital lessons for resource-constrained settings^3^ ^,^ ^4^ ^,^ ^9^. This emphasizes that technological integration must be culturally and structurally aligned.

## CONCLUSIONS

Prompt Engineering is more than a technical skill. It is a new pedagogy that forces us to be more critical thinkers. The quality of the AI’s response is a direct reflection of the clarity of human thought. While AI can process vast amounts of data, human clinical judgment remains the final and irreplaceable filter. As adapted from the principles of William Osler, the success of medical education lies not merely in the transfer of facts, but in forging a mind capable of interrogating reality with precision. Those who fail to embrace AI will be at a disadvantage, but those who trust it blindly will be at risk.

## DATA AVAILABILITY

The datasets generated and/or analyzed during the current study are available from the corresponding author upon reasonable request.
